# Behavioral effects of different contraceptive methods and HIV acquisition: an ancillary study of the ECHO randomized trial

**DOI:** 10.1186/s12978-021-01232-6

**Published:** 2021-09-29

**Authors:** Mandisa Singata-Madliki, Theresa A. Lawrie, Yusentha Balakrishna, Florence Carayon-Lefebvre d’Hellencourt, G. Justus Hofmeyr

**Affiliations:** 1 Effective Care Research Unit, Frere Maternity Hospital, Eastern Cape Department of Health/Universities of the Witwatersrand and Fort Hare, P.O. Box 4097, East London, South Africa; 2 FHI 360, 359 Blackwell St, Suite 200, Durham, NC 27701 USA; 3 Evidence-Based Medicine Consultancy Ltd, Bath, UK; 4 Biostatistics Research Unit, South African Medical Research Council, Durban, South Africa; 5 Department of Obstetrics and Gynaecology, University of Botswana, Botswana and Walter Sisulu University, East London, South Africa

**Keywords:** Contraception, HIV, Sexual behavior, DMPA, Intrauterine device, LNG implant, Condom, Menstruation

## Abstract

**Background:**

The ECHO trial randomised 7829 women to depot medroxyprogesterone acetate (DMPA-IM), the copper intrauterine device (IUD) and the levonorgestrel (LNG) implant (1:1:1) and found no clear difference in HIV incidence between these three groups. We have previously hypothesized that oligo-amenorrhoea induced by DMPA-IM may have a protective effect on HIV acquisition. The aim of this ancillary study was to assess the effects of DMPA-IM, the IUD and the LNG implant on menstrual symptoms and sexual behavior and to correlate these with HIV acquisition.

**Methods:**

At the Effective Care Research Unit (ECRU) in South Africa, of 615 women already randomised to DMPA-IM, the copper IUD and the LNG implant (1:1:1) 552 agreed to participate. Participants completed a 28-day symptom and behavior diary following their one-month ECHO trial visit and returning it at their 3-month follow-up visit. HIV acquisition data were retrieved from ECHO trial records.

**Results:**

Of 552 women enrolled on the ancillary study, 390 (70.6%) completed their daily diary; 130, 133, and 127 received DMPA-IM, IUD, and LNG implant, respectively. Thirty-three (5.9%) of these women acquired HIV. Women on the progestin-only contraceptives were more likely to experience amenorrhoea, as expected, and were less likely to have intra-menstrual coitus than IUD users (p < 0.001 for DMPA-IM vs IUD and p = 0.002 for implant vs IUD). Overall coital frequency was highest and condom usage lowest among DMPA-IM users. Intra-menstrual coitus correlated positively, and duration of menstruation correlated negatively, with HIV acquisition, although these effects were not statistically significant (p = 0.09 and p = 0.079, respectively).

**Conclusions:**

Findings support the hypothesis that oligo-amenorrhoea and the associated reduced intra-menstrual coitus may mitigate the potential for an increased biological risk of HIV acquisition with DMPA-IM but more evidence is needed.

*Study registration number* PACTR201706001651380

## Plain Language Summary

There have been concerns that the depot-medroxyprogesterone acetate injection (DMPA-IM) may increase the risk of getting HIV infection. However, a large multicenter randomized study, the ECHO trial, recently compared HIV incidence among women randomized to DMPA-IM, the copper intrauterine device (IUD) and the levonorgestrel (LNG) implant and found little difference in HIV risk between these methods. DMPA-IM often causes no or scanty menstruation; we hypothesized that this may have a protective effect on getting HIV, by reducing exposure to HIV during menstrual bleeding.

This ancillary study was done among ECHO trial participants at one of the ECHO study sites in South Africa. The aim was to assess the effects of the three different contraceptives on menstrual symptoms and sexual behavior and to correlate these with the risk of getting HIV. The study required women to complete a 28-day daily symptom and behavior diary after their one-month ECHO trial follow-up visit.

We found that fewer women had sex during their periods with DMPA-IM and the LNG implant than the copper IUD, probably because no or scanty menstruation is more common with both DMPA-IM and the implant. Although effects were not statistically significant, having sex during periods tended to have a higher risk of getting HIV and longer periods indicated a lower risk of getting HIV.

We concluded that sexual behavior related to menstruation may influence HIV acquisition and may partially explain why the ECHO trial found little difference in HIV incidence between the three contraceptives assessed despite observational evidence of higher biological risk with DMPA related to immune suppression.

## Background

In Sub-Saharan Africa, injectable progestin contraception remains the most popular form of contraception, accounting for 45% of modern method use [1]. However, there are concerns about the safety of intramuscular depot medroxyprogesterone acetate (DMPA-IM), in particular, amid high rates of HIV infection in this region [2]. Immunological and animal studies suggest an increased HIV susceptibility with medroxyprogesterone acetate, [3–6] supported by observational studies and meta-analyses suggesting an association between DMPA-IM and HIV incidence [2, 7, 8]. However, randomised clinical trials (RCTs) have failed to show a statistically significant increase in HIV acquisition with DMPA-IM compared with the copper intrauterine device (IUD) or the levonorgestrel (LNG) implant [9, 10]. Most recently, the Evidence for Contraceptive Options and HIV Outcomes (ECHO) Trial Consortium reported HIV acquisition rates for DMPA-IM, the copper IUD and the LNG implant of 4.19, 3.31 and 3.94 per 100 woman-years, respectively, and these differences were not statistically significant [10]. As a result, the World Health Organization has upgraded the medical eligibility status of DMPA-IM for women at risk of HIV acquisition from a Category 2 to a Category 1 (a condition for which there is no restriction for the use of the contraceptive method) [11].

The role of sexual behavior in relation to contraception and HIV acquisition is not known. We have previously hypothesized that DMPA-induced oligo-amenorrhoea may have a protective effect on HIV acquisition by reducing sexual exposure during menstruation, thereby mitigating any potential biological increase in HIV risk [12, 13]. Unprotected coitus during menstruation may well be a risk factor for HIV transmission; [14–18] however, little research has been done on this topic. In the ECHO trial, significantly less coitus during menses was reported among DMPA-IM users and LNG implant users than copper IUD users (p < 0.0001), [10] which according to our hypothesis, may be the mechanism that equalizes HIV acquisition across these different methods. ECHO trial data were based on recall rather than daily diary methodology.

Thus, the objective of this exploratory ancillary study to the ECHO trial was to compare the effects of DMPA-IM, the copper IUD and the LNG implant on intra-menstrual coitus and condom use, through the use of a daily symptom and behavior diary, and to correlate these with HIV incidence. We hypothesized that the progestin-only contraceptives would be associated with fewer events of coitus during menses, due to less menstrual bleeding, and more condom use, compared with the copper IUD; and that intra-menstrual coitus would be associated with an increased risk of HIV acquisition.

## Methods

### Design and participants

This prospective study involved ECHO trial participants at the Effective Care Research Unit (ECRU) in East London, South Africa, one of the ECHO trial participating centres. The ECHO trial recruited women who were sexually active, HIV sero-negative between the ages of 16 and 35 years who were seeking effective contraception and compared the effect of three contraceptive methods (DMPA-IM, the copper IUD and LNG implant) on HIV incidence and pregnancy rates [10]. Participating women at ECRU were additionally asked to take part in this ancillary study, which had two objectives. The first was to determine whether there was a difference in depression and sexual functioning between the three contraceptive methods; these findings have been published separately [19]. The second, and the last objective of this paper, was to determine whether there were differences between contraceptive methods in daily symptoms and behaviors that could explain patterns of HIV acquisition.

### Procedure, outcomes, and instruments

Following online participant registration, the ECHO trial centrally randomised 7829 women to DMPA-IM (150 mg/mL, Depot-Provera^®^, Pfizer) given intramuscularly every 12 weeks, insertion of the subdermal LNG implant (150 mg, Jadelle^®^, Bayer Schering Pharma) or insertion of the Copper IUD (Paragard-T 380A^®^, Duramed). Of the 615 women that were recruited to the ECHO trial at ECRU, 552 agreed to participate in the daily diary ancillary study. Participants enrolled at the one-month follow-up visit were given a 28-day daily symptom and behavior diary to complete from the day of their enrolment visit and were asked to return it to research staff at the 3-month ECHO trial visit. To optimize compliance and the quality of self-reporting, research staff assisted participants to complete their ‘day 1’ entries. Participants were contacted daily by phone and reminded to complete their diaries. Parameters measured in the daily diary included sexual interest, sexual activity, condom use, mood and characteristics of menstruation (Table 1). Data on HIV acquisition were retrieved from ECHO trial records.

**Table 1:** Daily diary questions

	Question	Answer
1	Have you had menstruation?	0 = none 1 = light 2 = normal 3 = heavy
2	Have you had menstruation pain?	0 = no menstruation 1 = no pain 2 = mild pain 3 = severe pain
3	Have you had sexual intercourse?	0 = no 1 = yes with steady partner 2 = yes with casual partner
4	If yes to question 3, was a condom used?	0 = no 1 = yes
5	Have you felt an urge to have sexual intercourse?	0 = no 1 = yes
6	Have you felt sad for no real reason?	0 = no 1 = yes
7	Did you feel that your partner loves you?	0 = no 1 = yes 2 = no partner

Please tell us what has happened from midday yesterday until midday today

### Statistics

A statistician (YB) from the South African Medical Research Council performed the statistical analysis using Stata Statistical Software V.15 [20]. Analysis was by intention-to-treat. Categorical outcomes were compared using the χ^2^ test unless events in any group were equal to or less than five, and then Fisher’s exact two-tailed test was used. Data were expressed as the proportion of positive responses (e.g., if number of menstrual days was 7 days out of 28, the proportion was 7/28 = 0.25); pooled data for each study group was then expressed as mean values (of the individual proportions or means) with SD and compared using the t-test if the data are normally distributed. For non-parametric data, we used the Wilcoxon test to compare medians and interquartile ranges. We conducted a logistic regression analysis to evaluate the effect of the different variables on risk of HIV acquisition and present these data as Odds Ratios (OR) with their 95% confidence intervals (CI).

## Results

Of the total sample of 615 women recruited at our centre to the main ECHO Trial, 552 agreed to participate in this daily diary study and 390 returned their daily diary with at least 27 completed days; 130, 133, and 127 received DMPA-IM, IUD, and LNG implant, respectively (Table 2). Demographic characteristics were similar between participants who completed the diary (n = 390) and participants who did not complete the diary (n = 162) (Table 3). Study interviews were conducted between September 2016 and March 2018. Characteristics of participants in the three study groups were comparable, and similar to the total sample.

**Table 2:** Participant daily diary completion

	DMPA-IM (n)	IUD (n)	LNG implant (n)
Enrolled in ECHO trial	205	205	205
Enrolled in daily diary study	188	180	184
Did not submit the diary	46	41	48
Completed 1 day	7	3	6
Completed 2 days	1	2	0
Completed 3 days	0	0	1
Completed 5 days	1	0	0
Completed 6 days	1	1	1
Completed 9 days	1	0	0
Completed 12 days	1	0	0
Completed 18 days	0	0	1
Completed 27 days	1	0	2
Completed 28 days	129	133	125

**Table 3:** Baseline demographics for the sample

Characteristic	Participants who did not complete the diary (n = 162)	Participants who completed the diary (n = 390)	Total (n = 552)	p-value
Age; mean (SD)	25 (5)	25 (4)	25 (4)	0.724
Highest education; n (%)				
Primary school, complete	1 (0.6)	1 (0.3)	2 (0.4)	0.519
Secondary school, not complete	80 (49.4)	213 (54.6)	293 (53.1)	
Secondary school, complete	67 (41.4)	143 (36.7)	210 (38.0)	
Attended post-secondary school	12 (7.4)	32 (8.2)	44 (8.0)	
Marital status; n (%)				
Never married	155 (95.7)	376 (96.4)	531 (96.2)	0.48
Married (monogamous)	4 (2.5)	11 (2.8)	15 (2.7)	
Separated	0 (0.0)	2 (0.5)	2 (0.4)	
Widowed	1 (0.6)	0 (0.0)	1 (0.2)	
Income source; n (%)				
None	136 (84.0)	335 (85.9)	471 (85.3)	0.595
Formal employment	8 (4.9)	13 (3.3)	21 (3.8)	
Self-employment	1 (0.6)	1 (0.3)	2 (0.4)	
Other	15 (9.3)	40 (10.3)	55 (10.0)	

### Menstruation and sexual intercourse

Women receiving DMPA-IM and the LNG implant were more likely to report having no menstruation during the 28 days of evaluation than those with the copper IUD (p < 0.001). There was a statistically significant difference between groups in the median number of days of menstruation, with women allocated to DMPA-IM and the LNG implant experiencing fewer bleeding days than those allocated to the copper IUD (p < 0.001) (Table 4).

**Table 4:** 28-Day daily symptom and behavior outcomes

Outcome	DMPA-IM n = 130	IUD n = 133	LNG implant n = 127	p-value		
				DMPA-IM vs LNG implant	DMPA-IM vs IUD	LNG implant vs IUD
No menstruation (%)	69 (53.1)	6 (4.5)	49 (38.6)	0.020	< 0.001	< 0.001
Menstruation duration in days median (IQR)	0 (0–4)	9 (5–14)	3 (0–9)	0.006	< 0.001	< 0.001
At least one day of severe menstrual pain (as a proportion of those reporting menstruation) n(%)	8/61 (13.1)	34/127 (26.8)	10/78 (12.8)	0.959	0.035	0.018
Sexual intercourse (days) mean [SD]	13.4 [9.3]	10.8 [7.8]	11.2 [8.7]	0.055	0.015	0.669
No sexual intercourse n (%)	11 (8.5)	9 (6.8)	10 (7.9)	0.864	0.604	0.732
At least one act of unprotected intercourse n (%)	89 (68.5)	79 (59.4)	74 (58.3)	0.074	0.394	0.356
Proportion of days with unprotected intercourse median (IQR)	0.11 (0–0.32)	0.07 (0–0.18)	0.04 (0–0.25)	0.039	0.013	0.724
Condom use with casual partners* (as proportion of sexual intercourse with a casual partner days) median (IQR)	1 (0.57–1)	1 (0.8–1)	1 (0.5–1)	0.918	0.365	0.337
Condom use with steady partners (as proportion of sexual intercourse with steady partner days)* median (IQR)	0.43 (0–1)	0.71 (0.33–1)	0.66 (0.13–1)	0.100	0.021	0.497
Intra-menstrual coitus (as a proportion of total days)* median (IQR)	0 (0–0.04)	0.07 (0–0.21)	0 (0–0.07)	0.135	< 0.001	< 0.001
At least one occurrence of intra-menstrual coitus(%)	48 (36.9)	86 (64.7)	58 (45.7)	0.154	< 0.001	0.002
Proportion of days with urge for sexual intercourse* (libido) median (IQR)	0.29 (0–0.64)	0.32 (0.14–0.57)	0.25 (0.04–0.5)	0.519	0.611	0.174
Proportion of days feeling partner does not love her* median (IQR)	0 (0–0.04)	0 (0–0)	0 (0–0)	0.964	0.872	0.861
Proportion of diary days feeling sad for no reason* median (IQR)	0.14 (0–0.50)	0.14 (0–0.36)	0.14 (0–0.39)	0.544	0.148	0.446

*Calculated per participant as a proportion of days with the occurrence and then calculated across each study group as mean [SD] or median (IQR). For example, condom usage would be calculated for each participant where the denominator is the number of days with sexual intercourse, such that 100% condom coverage would be 1

Thirty participants recorded having no sexual intercourse during the 28-day period. These participants were equally distributed across the groups (p = 0.872). Women using DMPA-IM were more frequently engaged in sexual intercourse than women using the copper IUD (p = 0.015) or the LNG implant (p = 0.055), although the latter difference did not reach statistical significance. DMPA-IM and LNG implant users (36.9% and 45.7%, respectively) were less likely to have intra-menstrual coitus than copper IUD users (64.7%) (p < 0.001 for DMPA-IM vs IUD and p = 0.002 for implant vs IUD).

The proportion of women experiencing at least one day of severe menstrual pain was statistically significantly higher in the IUD group compared with DMPA-IM and the LNG implant, p = 0.035 and 0.018, respectively.

### Condom usage

The proportion of coital events covered by condom usage was significantly lower amongst participants using DMPA-IM (vs IUD, p = 0.013; vs LNG implant, p = 0.039). However, there were no significant differences between the groups in the proportion of women having at least one incident of unprotected coitus (Table 4). There was no statistically significant difference between the study groups in the use of condoms with casual partners; however, IUD users were more likely to use condoms with steady partners than DMPA-IM users (p = 0.012).

### Libido

There was no difference in libido (the proportion of days that women had an urge for sexual intercourse) or other parameters measured (Table 4).

### Risk factors for HIV acquisition

Thirty-three women (8.5%) among our ancillary study cohort of 390 participants acquired HIV during the course of the ECHO trial. This incidence is similar to that of the larger cohort of 615 women who were recruited at ECRU for the ECHO trial. On logistic regression (n = 389), the number of occurrences of intra-menstrual coitus correlated positively with HIV acquisition (OR 1.11, 95% CI 0.97–1.26, p = 0.142), and duration of menstruation correlated negatively with HIV acquisition (OR 0.91, 95% CI 0.82–1.02, p = 0.109); these associations did not reach statistical significance (Table 5). No other variables were statistically significant.

**Table 5:** Risk factors for HIV acquisition

Risk factor*	Odds ratio	95% CI	p-value
Number of occurrences of intra-menstrual coitus	1.11	0.97–1.26	0.142
Number of occurrences of unprotected intercourse	1.03	0.96–1.09	0.402
Duration of menstruation	0.91	0.82–1.02	0.109
Number of occurrences of intercourse with a casual partner	1.06	0.98–1.16	0.159
Number of occurrences of intercourse with a steady partner	0.97	0.92–1.04	0.419

*CI* confidence interval

*Controlled for age, highest education, income source and contraceptive arm

## Discussion

This prospective study was ancillary to the large multicentre ECHO trial, a rigorous RCT that found no clear difference in HIV incidence among women randomised to DMPA-IM, the copper IUD and the LNG implant. Three hundred and ninety women in this ancillary study completed 27 or 28 days of their daily diaries and we used logistic regression to investigate behavioral risk factors for HIV acquisition using these data. The overall incidence of HIV in our cohort (8.5%) was higher than that of the main ECHO trial (5%), highlighting the enormous risk of HIV infection among contraceptive users in our South African setting.

As expected, oligo-amenorrhoea, a well-known side effect of progestin-only contraceptives, was much more common among DMPA-IM and LNG implant users than IUD users. As an apparent consequence of this outcome, intra-menstrual coitus was significantly more common among copper IUD users than users of the progestin-only methods. This was also a finding of the main ECHO trial, in which women were asked to recall at each follow-up visit whether they had had sexual intercourse during menses in the preceding 3 months [10]. Among ECHO trial participants, the frequency of intra-menstrual coitus (reported as 7.2%, 9.1% and 7.1% for DMPA-IM, the copper IUD, and the LNG implant, respectively), was much lower than that reported in our study, in which almost half of the participants had at least one episode of intra-menstrual coitus during the 28 days of study. This difference is probably a result of the different methodology employed, as recall in our study was a daily reflection, as opposed to recall in the main ECHO trial, which involved reflection over the preceding 90 days. However, the relative effects found in the ECHO trial were in the same direction, and statistically significant for DMPA-IM vs the copper IUD (p < 0.0001), as our study.

In our study, DMPA-IM users had the highest rates of amenorrhoea, the shortest duration of menstruation, and the lowest frequency of intra-menstrual coitus, probably because most women in this group experienced no menstruation during the study period. Conversely, copper IUD users had the lowest rates of amenorrhoea, the longest duration of menstruation and the highest frequency of intra-menstrual coitus. DMPA-IM users also had the highest coital frequency, which could be due to less days of menstrual bleeding and, therefore, more days available for coitus outside of menses. Higher rates of dysmenorrhoea may also have contributed to the lower coital frequency seem amongst copper IUD users in our study.

The main ECHO trial found no difference in self-reported coital frequency between groups [10]. However, as mentioned above, these findings were based on recall of sexual activity over the past 90 days, which is likely to be less accurate than daily recall of events from the preceding 24 h.

DMPA-IM users in our study were less likely to use condoms than copper IUD and LNG implant users. This may be because condoms were more likely to be used when coitus occurred during menstruation. Copper IUD users had the highest rate of condom use with steady partners. Again, this may be because menstruating women were more likely to use condoms, and copper IUD users had more intra-menstrual coitus than the other groups.

Our findings on reported condom use differ from those of the main ECHO trial, however, which found higher condom usage amongst the DMPA-IM users [10]. This difference may be due to behavioral differences between the study sites, but could also be a function of recall bias that might have occurred in the main ECHO trial in relation to this outcome.

Whilst no statistically significant associations with potential risk factors were found, the odds of HIV acquisition was increased with the occurrence of intra-menstrual coitus and reduced with longer duration of menstruation. Intra-menstrual coitus occurred in 49% of our study sample, indicating that this practice is common in our population. This is higher than found in another South African study that reported a lifetime incidence amongst 531 women of coitus during genital bleeding (80% of which was menstrual) of 26% [16]. Women in this study who reported the latter were over four times more likely to have been diagnosed with a sexually transmitted infection (STI) including HIV [16]. Similarly, a North American study reported an incidence of intra-menstrual coitus of around 26% among 1586 women, finding a “strong statistical association” of intra-menstrual coitus and self-reported STI history, and suggesting that this practice might emerge as a risk factor for heterosexual transmission of HIV [17]. Another study of HIV risk factors among women in the Dominican Republic reported that engaging in intra-menstrual coitus was associated with a three-times increased risk (OR 3.2) of being HIV positive [18]. In addition, other studies have linked sexual exposure during menstruation with an increased risk of gonococcal infection [21, 22].

Our finding of a tendency to a negative association between menstruation and HIV acquisition suggests that menstruation might be a protective factor for HIV acquisition, probably because women are less likely to engage in sexual intercourse during their menstrual periods and therefore have less sexual intercourse overall compared with women who do engage in intra-menstrual coitus. Women (and men) may intuitively feel more exposed to the risk of STI acquisition during menstruation and may prefer to avoid intra-menstrual coitus. In addition, decision-making about condom use during menses may be influenced by the desire to avert acquisition or transmission of STIs [23]. Reduced coitus and increased condom use during menses could also be related to an aversion to blood contamination, or for cultural or religious taboos; [24, 25] avoidance of intra-menstrual coitus is common to the teachings of all major religions [26].

The United Kingdom’s National Health Service advises using a condom for sex during menstruation to prevent HIV and other STIs [27]. However, more needs to be done globally to convey the importance of dual protection, especially during menstruation, and particularly where HIV and other STI rates are extremely high, as in our setting.

This ancillary daily diary study has several strengths: it was conducted alongside a rigorous RCT in which participants had been randomly assigned to contraceptive methods, therefore selection bias is unlikely to be responsible for behavioral differences. There were similar numbers of participants in each intervention group with similar baseline characteristics to the study center cohort; therefore, bias due to selective participation is unlikely. In addition, recall bias was minimized through the use of a daily symptom and behavior diary.

However, it also has several limitations. We correlated HIV acquisition during the course of the ECHO trial with a daily diary commenced and completed during the second month after enrolment; a subsequent follow-up diary would have been preferable to confirm these findings. In addition, the lack of significance in the findings on intra-menstrual coitus may have been due to the small sample size.

## Conclusions

These findings support the hypothesis that oligo-amenorrhoea and the associated reduced intra-menstrual coitus may mitigate the potential for an increased biological risk of HIV acquisition with DMPA-IM. In addition, this study adds to the small body of evidence that suggests there may be an increased risk of HIV infection with intra-menstrual coitus. Education about the potential increased risk of acquiring HIV by engaging in coitus during menstruation should be considered, with condom use stressed as critical during this phase of the menstrual cycle in particular. More research into risk factors for HIV acquisition among contraceptive users is needed.

## Data Availability

The datasets used and/or analysed during the current study are available from the corresponding author on reasonable request.

## Abbreviations

DMPA-IM: Depot medroxyprogesterone acetate injection
ECHO: Evidence for Contraceptive Options and HIV Outcomes
IUD: Intrauterine device
LNG: Levonorgestrel
STI: Sexually transmitted infection
